# Assessing Salmonella Typhi Pathogenicity and Prevention: The Crucial Role of Vaccination in Combating Typhoid Fever

**DOI:** 10.3390/ijms26093981

**Published:** 2025-04-23

**Authors:** Elena Roxana Buzilă, Olivia Simona Dorneanu, Felicia Trofin, Cristina Mihaela Sima, Luminița Smaranda Iancu

**Affiliations:** 1 Microbiology Discipline, Preventive Medicine and Interdisciplinarity Department, University of Medicine and Pharmacy “Grigore T. Popa”, 700115 Iasi, Romania; elena-roxana.buzila@umfiasi.ro (E.R.B.); felicia.trofin@umfiasi.ro (F.T.); cristina.sima@umfiasi.ro (C.M.S.); luminita.iancu@umfiasi.ro (L.S.I.); 2 Iasi Regional Center for Public Health, National Institute of Public Health, 700465 Iasi, Romania; 3 Clinical Hospital of Infectious Diseases “Sf. Parascheva”, 700116 Iasi, Romania; 4 “Sf. Spiridon” Clinical Emergency Hospital, 700111 Iasi, Romania

**Keywords:** *Salmonella* Typhi, pathogenicity factors, antibiotic resistance, typhoid fever, typhoid vaccine

## Abstract

Enteric fever is caused by *Salmonella enterica* serovar Typhi (*S.* Typhi) and *Salmonella enterica* serovar Paratyphi (*S.* Paratyphi) A, B, and C. Globally, an estimated 11 to 21 million cases of typhoid and paratyphoid fever occur annually, with approximately 130,000–160,000 deaths, most of which are reported in South/Southeast Asia and sub-Saharan Africa. The antibiotic susceptibility of *S.* Typhi strains varies between countries within broad limits, from 3% to 97% for ampicillin, 9% to 95% for ciprofloxacin, 4% to 94% for chloramphenicol (India vs. Pakistan), and 0% to 99% for ceftriaxone (India vs. Iraq). With *S.* Typhi increasingly exhibiting resistance to antibiotics, vaccination becomes an essential preventive measure. Currently, three vaccines are licensed for typhoid fever: the typhoid conjugate vaccine (TCV), live-attenuated oral vaccine Ty21a (Ty21a), and Vi capsular polysaccharide vaccine (Vi-CPS). While no specific vaccine exists for paratyphoid fever, the genetic and antigenic similarities between *S.* Paratyphi and *S.* Typhi offer potential for the development of such a vaccine. Early studies show promising results, demonstrating both safety and immunogenicity in preclinical trials. Whole genome sequencing (WGS) provides a powerful tool for assigning genotypes, identifying plasmids, comparing genetic elements, and investigating molecular factors that contribute to antibiotic resistance and virulence.

## 1. Introduction

Enteric fever, one of the foremost bacterial infections worldwide, is a common infection in regions with poor economic development and with a limited public health infrastructure. It continues to represent a significant public health burden in many low- and middle-income countries that lack safe drinking water and improved sanitation [1,2,3].

Human infection is predominantly caused by *Salmonella enterica* serovars Typhi (*S.* Typhi). The serovars of *S.* Typhi, Paratyphi A, Paratyphi B, and Paratyphi C, are collectively referred to as typhoidal *Salmonella* serovars, owing to the similarity in clinical manifestations observed in infections caused by Paratyphi strains and those caused by *S.* Typhi [1,2]. In the late 19th century, the United States (U.S.) began to experience a decline in the incidence of numerous infectious diseases, including typhoid fever, due to improvements in sanitation infrastructure—a trend that continued well into the 20th century [4]. According to the Global Burden of Disease (GBD) study conducted in 2017, the global incidence of typhoid and paratyphoid fever decreasing from 25.9 million cases in 1990 to 14.3 million cases in 2017 [5]. The incidence of typhoid fever appears to have remained relatively stable in recent years [6]. An accurate estimation of the global incidence of typhoid fever is limited by the fact that the diagnostic gold standard requires the isolation of *S*. Typhi through culture from a clinical sample [3].

It is estimated that between 11 to 21 million cases of typhoid and paratyphoid fever occur globally each year, resulting in approximately 130,000 to 160,000 deaths. The majority of these cases are concentrated in South/Southeast Asia and sub-Saharan Africa. Data from 2017 showed the highest incidence in India and Bangladesh, with about 500–700 cases per 100,000 of the population. In contrast, typhoid and paratyphoid fever are considered relatively rare in the European Union and European Economic Area (EU/EEA). [7], while in the US, there are approximately 5700 cases and 620 hospitalizations each year. Most people from these regions are infected during international travel, particularly to regions such as Southeast Asia, West and East sub-Saharan Africa, and Oceania [8,9]. Risk estimates for travelers are generally reported as incidence proportions (notifications per 100,000 travelers) rather than incidence rates (notifications per 100,000 person-years [PY]) [9]. Thus, the incidence of typhoid among travelers to selected low- and middle-income countries was ≤1 case per 100,000 travelers, with the exception of those who visited Nepal or India, where reported rates were 7.9 and 27–81 cases per 100,000 travelers, respectively [10].

### 1.1. Motivation

*S.* Typhi, the causative agent of typhoid fever, remains a significant public health challenge, particularly in low-resource settings where access to clean water and sanitation is limited. Despite the availability of antibiotics, the emergence of drug-resistant strains complicates treatment and highlights the urgent need for a deeper understanding of this bacterium’s pathogenic mechanisms in order to develop more effective preventive measures, particularly vaccines.

This comprehensive narrative review of *S.* Typhi’s pathogenesis and prevention strategies serves multiple purposes, offering valuable insights for researchers, healthcare professionals, and policymakers. It will provide a detailed examination of the bacteria’s biology, virulence factors, and its interactions with the host immune system. Given the growing prevalence of multidrug-resistant *S.* Typhi strains, there is an increasing emphasis on non-antibiotic preventive strategies, particularly vaccination. This review will critically assess current prevention approaches, identify gaps in the existing framework, and propose future research directions.

Vaccination remains a key tool for controlling typhoid fever in endemic regions, and this review will highlight recent advances in vaccine development, evaluate the effectiveness of the available vaccines, and explore the challenges related to vaccine implementation, all of which are essential for reducing the disease burden.

### 1.2. Aim

The objective of this narrative review is to provide an extensive analysis of the pathogenic mechanisms of *S.* Typhi, focusing on its virulence factors, invasion tactics, survival strategies within the host, and immune evasion methods, in order to more efficiently design preventive measures. For this reason, we will also assess current prevention strategies, including improvements in sanitation, hygiene practices, and access to safe water, as well as medical interventions such as antibiotic therapy and vaccination. A detailed evaluation of the existing vaccines (e.g., TCV, Ty21a, Vi-CPS) will be provided, covering their efficacy, safety profiles, and limitations. This review will also explore advancements in the development of new vaccines, particularly those aimed at addressing drug-resistant strains. Furthermore, recommendations for public health policies will be offered, with an emphasis on integrating vaccination programs in endemic regions, targeting vulnerable populations, and supporting global efforts to control the spread of typhoid fever.

## 2. Host, Transmission, Risk Factors, and Evolution

### 2.1. Host

The reservoir of *S.* Typhi is the human host [11,12]. Although the bacterium can persist in the environment for extended periods—ranging from 14 to 140 days [11,13,14]—it does not replicate in water or food sources [11]. *Salmonella*’s ability to persist outside an animal host is primarily due to its capacity to form biofilms, which provide a protective, nutrient-rich environment [13].

### 2.2. Transmission

The transmission of *S.* Typhi primarily occurs through the digestive route, often through the consumption of food or water contaminated with the feces or urine of infected individuals or asymptomatic carriers [11,12,15]. There are two primary pathways for *S.* Typhi transmission: the short cycle and the long cycle. The short cycle involves the contamination of food and water through the shedding of the bacteria by temporary or chronic carriers, a process facilitated by poor hygiene and sanitation, particularly during food handling. The long cycle occurs when human feces contaminate unsafe or untreated water sources, or when raw feces are used as a fertilizer for crops [11,12]. This type of transmission is challenging to trace, as *S.* Typhi is difficult to isolate from contaminated environments [11,16].

### 2.3. Risk Factors

The risk of *S.* Typhi’s transmission is higher in communities lacking access to safe water, adequate hand-washing practices, good hygiene, and effective waste management or where transmission occurs via houseflies carrying pathogens from human waste to food [12,15,17,18]. The consumption of street-vended foods, including dairy products, ice cream, fruits, and juices, is a well-established risk factor for typhoid fever [17,19]. Other contributing risk factors include a high population density, low socioeconomic status, low literacy rates, and occupational exposure, such as handling the bacteria in clinical microbiology laboratories [12,17]. However, infection can occur even in the absence of a clear association with population density [12].

The risk of developing typhoid fever varies across different age groups and genders [20] and is further influenced by underlying health conditions [21]. Among genetic factors, the HLA-B*27:05 allele has been strongly associated with an increased susceptibility to enteric fever, as it facilitates enhanced intracellular replication of *S.* Typhi [22].

The incidence of infection is low during the neonatal period and increases with age, likely due to the protective effect of maternal antibodies and limited exposure to contaminated food sources, as nutrition during this stage predominantly or exclusively relies on breastfeeding [23]. After this period, incidence rates increase among children, peeking in the 5–9-year age group and then steadily declining into adulthood [5]. Women and children under 15 represent the populations at greatest risk [15,20].

Travel to regions where typhoid fever is endemic, such as sub-Saharan Africa and Southeast Asia, is a significant risk factor. In these settings, the likelihood of contracting the infection increases with the length of stay, with the disease being detected in about 20% of vaccinated travelers who stayed for less than a month, compared with almost 35% among those who spent at least six months in these areas [24].

However, data suggest that typhoid fever is a risk even for short-term travel. About 5% of U.S. travelers diagnosed with typhoid fever reported being out of the country for less than a week, and 60% reported a travel duration of less than six weeks [25]. Other travel-related risk factors include the purpose of the trip and the type of accommodation. But there are also intrinsic risk factors such as age and diagnosed/undiagnosed underlying medical conditions [10].

*S.* Typhi enters the body via the digestive tract, usually by ingesting water or food contaminated with the feces of infected patients or symptomatic carriers of the bacteria [11,15,23]. Gastric acid serves as an important barrier to *S.* Typhi, inhibiting its penetration of the small intestinal mucosa and subsequent dissemination to the reticuloendothelial system [23]. The infective dose of enteric pathogens is typically influenced by various factors, including their ability to withstand acidic conditions [24]. Clinical studies suggest that the infectious dose (ID) for *Salmonella* is at least 10⁵ bacteria, but outbreak investigations have shown that illness can result from ingesting as few as 50–100 organisms, particularly via contaminated food [26]. Furthermore, certain solid food sources, particularly those high in fat or protein, are thought to protect *Salmonella* from the acidic conditions in the stomach [27].

### 2.4. Evolution

The bacterium is primarily excreted in feces beginning in the first week of infection, with the highest probability for isolation starting from the second week and, in urine, starting from the third week. It can also serve as a vehicle of transmission during both acute and subclinical infections [23,27,28,29]. Shedding may either be transient or chronic. Transient shedding can occur during the acute or convalescent phases, with convalescent carriers shedding *S.* Typhi for 3 to 12 months after an acute infection. Chronic carriers continue to excrete the bacteria for over 12 months after the initial infection [23]. Chronic asymptomatic carriers play a crucial role in maintaining the presence of *S.* Typhi in human populations. Since most carriers in endemic regions show no symptoms and may have no prior history of typhoid fever, the condition remains under-researched. However, it is recognized that chronic carriage is more prevalent in women and increases with age [10,23]. Chronic carriers are frequently associated with persistent gallbladder infections [30,31], with gallstones present in approximately 90% of cases, significantly increasing the risk of gallbladder cancer [31]. Biofilm formation on gallstones is strongly correlated with asymptomatic bacterial carriage [10,32]. Additionally, the liver may serve as a reservoir for chronic infections, periodically releasing bacteria into the gallbladder, which is a primary source for fecal excretion [31,33]. Typhoid and paratyphoid fever are clinically difficult to differentiate. The incubation period typically ranges from 8 to 14 days, but may vary between 3 and 60 days [34], with this variability mainly dependent on infective dose and host immune response. The clinical presentation of enteric fever is diverse and can resemble other systemic conditions, including fever, chills, headache, anorexia, abdominal discomfort, relative bradycardia, vomiting, diarrhea, constipation, hepatomegaly, splenomegaly, leukopenia, and thrombocytopenia [1]. The illness duration can range from a few weeks to several months, depending on the initial infectious dose, as well as the timing of diagnosis and treatment. These factors also determine the severity of the disease and its potential complications [34]. During the course of infection, the bacteria disseminate to the gallbladder, liver, and spleen [31].

Enteric fever progresses through two phases: an initial phase characterized by low-grade fever (37.5–38.2 °C), which escalates to high-grade fever (38.2–41.5 °C) in the second week. In untreated cases, fever can persist for a month or longer and is often accompanied by bradycardia, myalgia, splenomegaly, hepatomegaly, and the appearance of pink spots on the abdomen and chest [31,33]. Common complications include anemia, gastrointestinal bleeding, intestinal perforation, bone marrow hypoplasia, encephalopathy, disseminated intravascular coagulation, and shock [1].

## 3. Pathogenicity Factors

### 3.1. Typhoid Toxin

Typhoid toxin is expressed by both typhoid serotypes, *S.* Typhi and *S.* Paratyphi, but is absent in other *S. enterica* serotypes associated with self-limited gastroenteritis [35,36]. It represents a key virulence factor in *S.* Typhi strains, capable of inducing many symptoms characteristic of typhoid fever when administered to experimental animal models [37]. Belonging to the AB-type bacterial toxin family, typhoid toxin comprises an enzymatic subunit “A” and a receptor-binding subunit “B”, and it is expressed exclusively by intracellular bacteria [35,37,38]. This toxin appears to have evolved through the functional convergence of two exotoxins: cytolethal distending toxin (CDT) and pertussis toxin [31]. In contrast to CDT, which is encoded by various microorganisms such as *Escherichia coli*, *Haemophilus*, and *Yersinia*, typhoid toxin possesses a unique A_2_B_5_ structural configuration. This structure includes two distinct “A” subunits (CdtB and PltA), homologous to the A subunits of CDT and pertussis toxin, along with a pentameric “B” subunit (PltB), homologous to the B subunit of pertussis toxin. All components of the toxin are chromosomally encoded [35,36,39,40]. Structurally, typhoid toxin forms a pyramid, with PltB at the base, PltA positioned centrally, and CdtB located at the apex [40]. PltA is anchored to the PltB oligomer via a short helix at its terminal end, which inserts into the hydrophobic lumen of the PltB channel. In contrast, CdtB exhibits minimal interaction with PltA and does not directly contact PltB [40]. A distinctive disulfide bond between CdtB (Cys269) and PltA (Cys214) represents an evolutionary adaptation that contributes to the unique functionality of typhoid toxin [39,40]. Additionally, PltA contains a third cysteine residue not found in its homologs, while CdtB possesses Cys269, a residue specific to typhoid toxin [40].

Since the toxin is produced exclusively by intracellular *S.* Typhi strains, its export to the extracellular environment is mediated by *Salmonella*-containing vacuoles (SCVs), enabling it to infect other target cells [38]. Upon binding to the target cell surface via the PltB subunit, the PltA and CdtB subunits are internalized. CdtB is subsequently translocated through the Golgi apparatus and endoplasmic reticulum to the nucleus, where its DNase-I nuclease function induces DNA damage, cell cycle arrest (in G1, S, or G2 phases), and eventually, cell apoptosis [36] (Table 1).

The genes encoding typhoid toxin are located within a genomic island that contains sequences homologous to the active subunit CdtB, as well as genes encoding PltA and PltB, the components of pertussis toxin [41,42]. The linear arrangement of this complex and the lack of interaction between CdtB and PltB explain why deletion of the pltA gene results in the loss of CdtB-mediated toxicity [39]. Mutations in pltA or pltB impair the functionality of the toxin complex, resulting in a complete loss of CdtB-dependent toxic activity, functionally linking these three genes. Biochemical studies have further confirmed that CdtB, PltA, and PltB collectively form an essential assembly for toxin activity [42].

Notably, typhoid toxin cannot toxify the host cells in which it is produced unless it is first released into the extracellular space. For example, adding a specific antibody to the extracellular environment effectively protects infected cells from toxification, supporting the hypothesis that typhoid toxin exerts its effects via a paracrine or autocrine mechanism [42].

### 3.2. Antigens of S. Typhi

**Figure 1:**
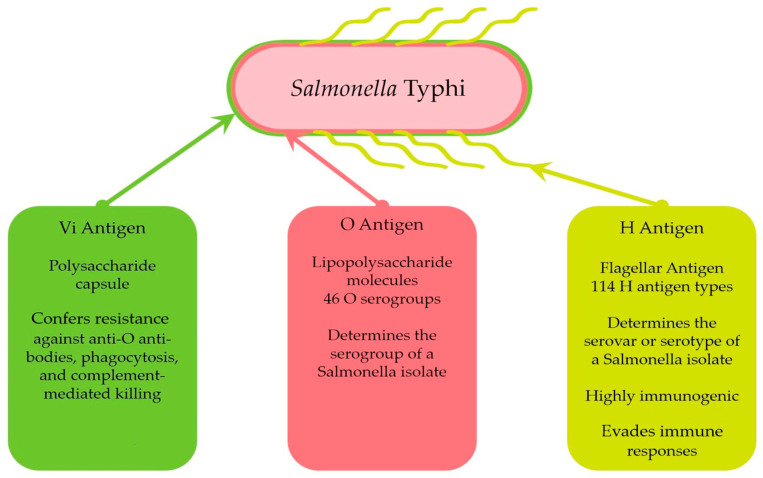
Description of antigens of *Salmonella* Typhi.

*S.* Typhi possesses three principals antigens: the H (flagellar) antigen, the O (somatic) antigen, and the Vi (virulence) antigen. Traditionally, *Salmonella* serovars are classified using agglutination-based methods that assess the antigenic variability in the O and H surface antigens, which correspond to lipopolysaccharide (LPS) and flagellin molecules, respectively [43]. The O antigen is used to determine the serogroup, while the H antigen completes the serovar or serotype identification of a *Salmonella* isolate [44]. The Kauffmann–White–Le Minor scheme describes 46 O serogroups [44] and 114 H antigen types [45] (Figure 1).

### 3.3. Flagellar Antigen—H Antigen

The H antigens of *Salmonella* are well characterized and are primarily encoded by two genes: *fli*C and *flj*B. These genes express the phase 1 and phase 2 H antigens, respectively. The *fli*C gene, located within one of the flagellar biosynthesis operons, is present in all *Salmonella* strains and has homologs in other enteric bacteria. Conversely, the *flj*B gene is located in a genomic region unique to *S. enterica* [46]. While flagellin is highly immunogenic, its variation among serovars enables *Salmonella* to evade host immune responses. This immune evasion occurs when a host previously exposed to one flagellar variant becomes infected with a strain expressing a different variant [47] (Figure 1).

### 3.4. Somatic Antigen—O Antigen

The thermostable somatic O antigen, located in the outer membrane of bacterial cells, represents the oligosaccharide component of LPS [48]. The genes responsible for O antigen synthesis are usually organized in a cluster on the chromosome between the *gal*F and *gnd* genes. Genetic variability within this gene cluster accounts for the differences among various O antigen types [44] (Figure 1).

### 3.5. Vi Antigen

A distinguishing feature of *S.* Typhi compared to non-typhoidal *Salmonella* (NTS) is the production of a polysaccharide capsule known as the Vi antigen. This capsule inhibits phagocytosis and confers serum resistance, likely by shielding the O antigen from antibody activity [49]. The Vi antigen’s expression is linked to resistance against anti-O antibodies, phagocytosis, and complement-mediated killing. However, these last two processes can be initiated by anti-Vi antibodies [50]. The resistance to killing correlates with the level of Vi antigen expression. The Vi capsule enhances the resistance of *S.* Typhi and *S.* Typhimurium strains to complement mediated and antibody-dependent killing by phagocytes in the presence of human serum [51].

The Vi antigen is a linear homopolymer of N-acetylgalactosaminuronic acid (D-GalNAcA), linked via α-1,4 linkages, with variable acetylation at the C-3 position. O-acetyl groups cover a significant portion of the surface, and the antigen’s immunogenicity is closely associated with its degree of acetylation [52]. Structurally, the Vi capsular polysaccharide is a homopolymer of (1,4)-2-acetamido-3-O-acetyl-2-deoxy-α-D-galacturonic acid, anchored to the outer membrane by a terminal N-acetylhexosamine residue modified with two β-hydroxy acyl chains [53].The biosynthesis of the Vi capsule in *S.* Typhi is encoded by the *tviB-E* and *vexA-E* genes, located in the *viaB* locus on the chromosome within *Salmonella* Pathogenicity Island-7 (SPI-7) [54]. This *viaB* locus, present in the *S.* Typhi genome but absent in *S.* Typhimurium, encodes genes for the regulation (*tviA*), synthesis (*tviBCDE*), and export (*vexABCDE*) of the Vi antigen. The *tviA* gene activates the synthesis and export genes for the Vi antigen, while suppressing genes involved in flagellar synthesis and the invasion-associated type III secretion system (T3SS). This suppression reduces inflammatory responses during intestinal mucosal invasion [55] (Figure 1).

### 3.6. Virulence Determinants That Facilitate Attachment

Biofilm formation is a critical factor in the persistence of *S. enterica*, especially within the gallbladder, where bile salts and inflammation create conditions conducive to long-term colonization. Notably, cholesterol-coated gallstones offer an optimal surface for biofilm development, thereby promoting the asymptomatic carriage of *S.* Typhi [56].

Multiple genes contribute to the formation and regulation of biofilms in *Salmonella*. Mig-14 is implicated in resistance to antimicrobial peptides and the modulation of membrane permeability. GalE is essential for the biosynthesis of LPS and colanic acid, both of which are important for biofilm stability. The QseB/QseC two-component regulatory system modulates the balance between biofilm formation and virulence. Additionally, several other genetic elements contribute to this process, including the long non-coding RNA AsfD and the non-coding RNA Ribs, as well as VirK, LuxS, Rck, and the plasmid pRST98 [56].

Flagella and fimbriae also play a role in initiating biofilm formation, attachment to host cells, and colonization [56,57]. Fimbriae are extracellular proteinaceous structures, typically measuring 0.5–10 μm in length and 2–8 nm in diameter, that play a pivotal role in bacterial adhesion—an essential initial step in the colonization and invasion of host tissues. Unlike flagella, fimbriae are not involved in bacterial motility; however, they contribute to interactions with macrophages, biofilm development, intestinal persistence, and bacterial aggregation [58,59]. By binding to specific receptor proteins on host cells, fimbriae facilitate intestinal colonization and the pathogenesis of salmonellosis. Moreover, they are capable of activating stromal fibroblasts, thereby promoting the secretion of extracellular matrix components that serve adhesive and structural functions [58].

*S*. Typhi encodes 12 distinct fimbriae-associated gene clusters. Experimental deletion of the *stg*, *bcf*, *saf*, or *stc* genes led to a significant reduction in biofilm formation, with levels decreasing to 70–85% of that observed in the wild-type strain. Experiments that had, as their purpose, supplementation with the same genes (*Stg*, *Sth*, *Bcf*, and *Ste*) also resulted in a reduced biofilm formation, reaching, in this case, 72–88% relative to the control. In contrast, introduction of the *Stb* gene enhanced biofilm production to 128%. Furthermore, overexpression of the *Fim*, *Stc*, *Std*, and *Tcf* genes increased biofilm formation to 140–180% compared to the control strain, while expression of *Stg* alone led to a further reduction, lowering biofilm formation to 68% [57,59,60].

Flagella play a dual role in *S.* Typhi, contributing not only to bacterial virulence but also to the activation of the innate immune system through the recognition of flagellin by Toll-like receptor 5 and NAIP receptors. In contrast to most NTS serotypes, which can alternately express two types of flagellin—FliC and FljB—*S.* Typhi is typically monophasic, expressing only the H:d antigen FliC [49]. Flagellin (FliC) is crucial for the initial adhesion to cholesterol-rich surfaces. Moreover, the absence of the outer membrane protein *Omp*C has been shown to impair biofilm formation [56]. Additionally, reduced flagellar expression in *S.* Typhi is associated with diminished inflammatory responses [61].

Within infected macrophages, flagellin is translocated into the cytosol via the T3SS-1, leading to the activation of inflammasome-mediated cell death through caspase-1 activation [57].

### 3.7. Virulence Determinants Affecting Intracellular Survival

Virulence factors are crucial for *Salmonella*’s intracellular survival. The bacterium enters host cells via phagocytosis, part of the innate immune response, or through active invasion mediated by T3SS-1. While phagocytosis targets a wide range of pathogens, T3SS-1-mediated invasion is a specific, tightly regulated process dependent on the coordinated expression of bacterial effector proteins [62].

T3SS is a specialized protein complex that translocates virulence factors into host cells, consisting of 20–30 structural and regulatory proteins. Key effectors, such as SipA, SipC, SopB/SigD, SopD, SopE2, and SptP, induce significant rearrangements of the host cell actin cytoskeleton, facilitating efficient bacterial internalization [62,63].

The deletion of any one of these has a minimal effect on invasion, whereas the deletion of two or more results in a significant invasion defect [62,63]. T3SSs have been viewed as promising targets for the development of anti-virulence agents, and some T3SS inhibitors have been identified [64].

Both T3SS-1 and T3SS-2 promote *S*. Typhi replication in human macrophages, and in addition, both T3SSs contribute to *S*. Typhi’s colonization of the spleen and liver in a humanized mouse model of typhoid fever [65]. T3SS-1 and T3SS-2 are encoded by SPI-1 and SPI-2, respectively [64].

The pathogenicity islands SPI-1 and SPI-2 contain numerous virulence genes involved in intracellular pathogenesis and encode T3SS, molecular mechanisms by which *Salmonella* transfers effectors into host cells [64].

SPI-1 encodes genes like *inv*, *hil*, *org*, *sip*, *spa*, *prg*, and others, which regulate the secretion of T3SS-1 effectors. These effectors are essential for the invasion of intestinal epithelial cells and the induction of necrosis and inflammation in macrophages [64]. The structural genes for the T3SS on SPI-1 include *prg*HIJK, *spa*MNOPQRS, and *inv*ABCEFGH, along with various regulatory and effector genes. HilA is the primary regulatory protein of the T3SS, and its expression is influenced by environmental factors essential for the bacterium’s survival [63].

SPI-2 encompasses more than 40 genes organized into four operons, including *ssa* (T3SS-2), *ssr* (regulator), and *ssc* (molecular chaperone). T3SS-2 facilitates the translocation of over 20 effectors into the host cell cytosol, playing a critical role in the survival and replication of the bacterium within phagocytes and epithelial cells during the intracellular phase of infection [64].

The virulence plasmids of *Salmonella* are collectively designated as pSV plasmids, which carry the spv operon that contains the *spv*R gene, encoding a LysR-type transcriptional regulator that positively controls its own expression, as well as that of the spvABCD operon. The SpvB, SpvC, and SpvD proteins are delivered into the host cell via a T3SS encoded by SPI-2 [66]. SpvB ADP-ribosylates actin, destabilizing the cytoskeleton and contributing to cytotoxicity, while SpvC inactivates MAP kinases, disrupting intracellular signaling in mammalian cells [57,66]. Although the *spv* virulence genes are present in *S.* Typhimurium, they can also be expressed in *S.* Typhi in an RpoS-dependent manner [67].

*S.* Typhi possesses several virulence factors, including LPS, a key component of the outer membrane of Gram-negative bacteria. As a potent endotoxin, LPS activates the immune response and induces nonspecific inflammation, particularly within the gastrointestinal tract, where it is often referred to as an enterotoxin. While endotoxins can cause severe effects such as fever, tissue necrosis, and lethality, they can also stimulate beneficial immune responses. Due to their central role in infection pathogenesis, endotoxins remain a critical focus in research on Gram-negative bacterial diseases [68].

## 4. Evolution of Antibiotic Resistance

Before the routine use of antibiotics, typhoid fever posed a significant challenge due to difficulties in the identification and selection of effective treatments. In the 1940s, chloramphenicol was the primary antibiotic used for treating typhoid fever; however, resistant strains of *Salmonella* spp. began to emerge [11].

A case report from the early 1950s described a 43-year-old patient diagnosed with typhoid fever who initially harbored a chloramphenicol-susceptible strain, which developed resistance during treatment [69]. Until the beginning of the 1960s, reports of chloramphenicol-resistant strains became more frequent [70,71,72]. By then, ampicillin and cotrimoxazole had been introduced as first-line treatments alongside chloramphenicol [73]. But the continued use of these antibiotics has accelerated the growth of resistance to these drugs [74]. However, simultaneous resistance to all three drugs, referred to as multidrug resistance (MDR), was first documented during a major outbreak in Mexico in 1972 [73].

Between the 1970s and 1990s, typhoid fever remained prevalent in regions lacking an adequate laboratory infrastructure to culture *S.* Typhi. Consequently, the prevalence of MDR strains was under-reported and likely underestimated [73]. Since the emergence of MDR strains in the 1980s, their prevalence has risen significantly. Initially, resistance targeted second-generation antibiotics, such as fluoroquinolones. Later, resistance expanded to include third-generation antibiotics, such as cefoperazone, cefotaxime, and ceftriaxone, as well as other cephalosporins [11]. Currently, fluoroquinolones and broad-spectrum cephalosporins are recommended for treating MDR *S.* Typhi infections due to resistance against traditional antibiotics [70,74].

The rising prevalence of antibiotic-resistant *S.* Typhi strains is strongly associated with the inappropriate use of antimicrobials, including self-medication, over-the-counter sales, and non-compliance with prescribed treatments. In low- and middle-income countries such as Nigeria, Pakistan, and India, factors such as insufficient pharmacy regulation, the availability of counterfeit antibiotics, and a lack of proper diagnostic procedures facilitate the spread of resistance genes, particularly against macrolides, fluoroquinolones, and β-lactams [74].

A significant contributor to the emergence of antibiotic resistance is the presence of counterfeit drugs, which may contain insufficient or no active ingredients, or may include harmful substances, and the use of counterfeit antibiotics, leading to a high number of false-positive reports [75]. Additionally, the over-prescription and prolonged, often empirical, use of antibiotics in the absence of blood cultures or an adequate healthcare infrastructure further exacerbate the development of resistance. Furthermore, antibiotics are frequently used to treat other infections (e.g., respiratory and skin infections), thereby promoting the selection of resistant bacterial strains [74].

In regions where enteric fever is endemic, laboratories often lack the capacity to accurately identify bacterial strains and perform antimicrobial susceptibility testing. As a result, knowledge of antimicrobial resistance (AMR) prevalence in *S.* Typhi and *S.* Paratyphi A strains remains limited. Data are often derived from individual hospitals and institutions, with limited comparability across regions and time periods. An improved understanding of AMR trends in enteric fever could support targeted public health measures, including vaccination campaigns and water, sanitation, and hygiene (WASH) interventions [76].

Surveillance studies reveal a significant geographic variation in *S.* Typhi susceptibility profiles and resistance trends, which differ by country. The evolution of antibiotic resistance in *S.* Typhi in endemic areas has progressed through distinct stages, characterized by the emergence of resistant strains and regional differences in susceptibility (Table 2). Between the 1960s and 1980s, strains resistant to cotrimoxazole and chloramphenicol were not reported [77,78,79,80], although resistance to streptomycin ranged from 50 to 100% [77,78]. In the 1980s, resistance to sulfonamides, trimethoprim, and chloramphenicol emerged [78,81,82]. In the 2000s, resistance to ciprofloxacin increased, reaching up to 95% in Bangladesh. Since 2017, susceptibility to first-line antibiotics such as amoxicillin and chloramphenicol has risen, with over 80% of strains showing susceptibility [83]. In India, resistance to ciprofloxacin has escalated, reaching up to 98%, while susceptibility to first-line antibiotics (chloramphenicol, ampicillin, and trimethoprim–sulfamethoxazole) has fluctuated [84,85,86,87,88]. Studies conducted in Kenya between 2004 and 2007 reported a high susceptibility to ceftriaxone (94%) and gentamicin (97%) but substantial resistance to ampicillin, chloramphenicol, and cotrimoxazole (70–72%) [85]. Additionally, 70% of the isolates were MDR [89]. Another study from the same region reported a reduced susceptibility to ciprofloxacin, with susceptibility rates of 20% in 2012 and 43% in 2013, while MDR rates were 79% in 2012 and 86% in 2013 [90]. Between 2005 and 2009, 66% of *S.* Typhi strains in India remained susceptible to first-line antibiotics, while 22% were classified as MDR [86]. From 2011 to 2020, susceptibility to these antibiotics increased to over 90%, and the proportion of MDR strains decreased to 2%. However, resistance to ciprofloxacin continued to rise, surpassing 95% by 2018 [87]. Epidemiological data on typhoid and paratyphoid fever remain limited, particularly in regions such as sub-Saharan Africa, Oceania, and Latin America. Nonetheless, the existing evidence indicates that the highest incidence rates are observed in South Asia, followed by Southeast Asia, Western and Eastern sub-Saharan Africa, and Oceania [5,10]. In contrast, East Asia reported relatively low AMR rates during the 2000–2018 period, with estimates as low as 3% [91]. Findings from the African Typhoid Surveillance Program and other research initiatives suggest that the incidence of typhoid fever in several African regions may equal or even surpass that of Asia, with significant impact observed in both rural and urban populations [10,92,93].

The limited data on the genetic diversity of *S.* Typhi in Latin America, where typhoid fever is endemic, combined with inadequate blood culture surveillance, hinder an accurate estimation of the disease burden [94]. In Colombia, 84% of *S.* Typhi isolates were susceptible to all tested antimicrobials, while 6.9% displayed resistance to ampicillin and 2.2% to ciprofloxacin [95].

Resistance to first-line antibiotics shows significant regional variation: 18–27% in Bangladesh, 2–3% in Nepal, and 82% in Pakistan. Resistance to ceftriaxone is reported at 65% in Pakistan but has not been observed in Bangladesh and Nepal. In all three countries, resistance to fluoroquinolones remains high [96]. In Pakistan, resistance to multiple antibiotics, including amoxicillin and fluoroquinolones, continues to rise, with the emergence of imipenem resistance raising significant public health concerns [97,98,99].

In Iraq (2019–2021), high resistance was observed to ceftriaxone (92%) and ampicillin (77%), but susceptibility to tetracycline, cotrimoxazole, and ciprofloxacin increased during the same period [100]. Conversely, in Ethiopia (2010–2021), susceptibility to ceftriaxone was high (94%), while it was low for chloramphenicol (11%) [101].

Typhoid fever presents significant treatment challenges, particularly in resource-limited settings where extensively drug-resistant (XDR) strains impose heavy healthcare and economic burdens. In Pakistan, treatment is often empirical, especially for adults, while children are more frequently hospitalized. The available therapeutic options are limited to azithromycin, meropenem, and piperacillin/tazobactam. However, premature treatment discontinuation increases the risk of complications. The rise of carbapenemase-producing *Salmonella* strains further undermines carbapenems’ efficacy, often leaving azithromycin as the only effective treatment [102].

In high-income regions with access to clean water and sanitation, such as Europe, Australia, and North America, typhoid fever is relatively rare, with incidence rates generally below 10 cases per 100,000 person-years. Most cases are travel-associated, particularly among individuals visiting friends and relatives in endemic areas [103]. Although the overall risk of typhoid fever remains low in developed countries like the U.S., the prevalence of drug-resistant *S.* Typhi strains is on the rise [104].

In Europe, travel to the Indian subcontinent is the leading risk factor for *S.* Typhi infection. In 1999, the Enter-net International Salmonellosis Database recorded 127,278 salmonellosis cases across EU member states and select non-EU countries, of which 461 (0.36%) were due to *S.* Typhi. Among cases with an available travel history (198 cases), 58% were associated with travel to the Indian subcontinent, followed by Papua New Guinea (9%), Indonesia (7%), and Tunisia (7%), with the remaining cases linked to various other countries [104].

In 2016, 22 EU countries reported over 1100 cases of typhoid and paratyphoid fever, with approximately 70% of cases concentrated in France, Italy, and the United Kingdom. Among patients with a recorded travel history, 83% had recently traveled internationally, primarily to India and Pakistan [105].

Between 1996 and 1997, a total of 293 cases of symptomatic typhoid fever were reported in the U.S., with the majority linked to recent travel to India, Pakistan, Bangladesh, or Haiti. Approximately 78% of the affected individuals required hospitalization, and two fatalities were recorded. Of the *S.* Typhi isolates obtained, 74 demonstrated AMR, including 51 classified as multidrug-resistant. Although the overall incidence of typhoid fever in the U.S. has remained relatively stable over the past two decades, there has been a notable rise in both the proportion of travel-associated cases—particularly to the Indian subcontinent—and the prevalence of multidrug-resistant strains. Individuals born outside the U.S. who travel to their countries of origin, along with children, represent the most vulnerable populations, underscoring the critical role of pre-travel vaccination for those visiting endemic regions [104].

Between 2008 and 2012, 2341 enteric fever cases were reported in the U.S., with 80% due to typhoid fever and 20% to paratyphoid A, the latter increasing from 16% to 22% over the period. The majority of cases (86%) were associated with recent travel, primarily to South Asia. AMR was common: 70% of *S.* Typhi isolates showed reduced fluoroquinolone susceptibility, and 12% were multidrug-resistant. While enteric fever remains rare in the U.S., travel-related cases dominate, mirroring resistance patterns in South and Southeast Asia. Typhoid vaccination is recommended for travelers, though no vaccine exists for paratyphoid fever [106].

Military personnel deployed to endemic regions also represent a vulnerable group. A review of typhoid fever cases reported in the U.S. Army from 1998 to 2011 documented 205 confirmed cases among active-duty service members, highlighting occupational exposure risks during overseas deployments [103].

The pathogen’s ability to develop resistance to multiple drugs has led to the emergence of MDR strains resistant to three first-line antibiotics, ampicillin and trimethoprim–sulfamethoxazole, and chloramphenicoland XDR strains, which exhibit resistance to five antibiotics: chloramphenicol, ampicillin, cotrimoxazole, fluoroquinolones, and third-generation cephalosporins. Currently, only three antimicrobial agents remain effective against XDR strains: oral azithromycin and the parenteral agents carbapenems and tigecycline [107]. XDR strains were first identified in Pakistan in 2016 [108], and since then, cases of XDR *S.* Typhi linked to travel to Pakistan have been reported in the Middle East, North America, Europe, Australia, and Taiwan [109].

These strains are challenging to treat, with only three antibiotics—azithromycin, carbapenems, and tigecycline—remaining effective. This situation severely limits treatment options and increases the risk of severe, life-threatening disease forms in affected patients [107].

**Table 2:** Evolution of antibiotic resistance for *Salmonella* Typhi.

**Year of Publication of Study**	Study Interval	Region	Batch Size	Evolution of Resistance Percentages												References
				AM %	AMX %	C %	CX %	NA %	CFM %	CRO %	SSS %	TE %	CIP %	STR %	IPM %	
1977	1965–1975	Jamaica	84	4–0	-	0	0	-	-	-	0	2–0	-	4–100	-	[77]
1987	1973–1982	Hong Kong	349	0	-	0	0	-	-	-	15	0	-	50	-	[78]
2020	1972–1989	South Africa	3327	1.1	-	0.1	-	0	-	-	-	-	-	-	-	[92]
2000	1975–1998	Tokyo	130	0–13	-	0–12	0–17	0	0	-	-	-	-	-	-	[82]
2020	1990–1999	South Africa	3327	39	-	52	35	12	-	-	-	-	-	-	-	[89]
2024	1999–2022	Bangladesh	12,435	-	60–20	40–20	40–20	-	-	-	-	-	56–98	-	-	[83]
2006	1999–2004	India	629	31–67	-	27–17	30–33	54–88	-	0	-	-	0–1	-	-	[84]
2020	2000–2018	Laos	913	8	-	8	-	-	-	-	-	-	1.4	-	-	[91]
2020	2000–2009	South Africa	3327	25	-	25	25	3	-	0.7	-	-	1.2	-	-	[89]
2018	2004–2007	Kenya	144	72	-	72	70	-	-	6	-	-	69	-	-	[85]
2012	2005–2009	India	337	25	-	23	31	73–84	-	0	-	-	0–9	-	-	[86]
2022	2011–2020	India	871	4	3	3	-	-	-	-	-	-	3–95	-	-	[87]
2020	2010–2018	South Africa	3327	23.	-	68	27	14.2	-	0.2	-	-	1.2	-	-	[89]
2019	2016	Ethiopia	14	100	100	100	-	-	-	64	-	79	-	-	-	[93]
2021	2017–2020	India	2032	3	-	4	4	-	0	0	-	-	98	-	-	[88]
2020	2012–2015	Columbia	402	7.5	-	0.7	1.7	5.7	-	-	-	3	2.2	-	-	[95]
2020	2016–2019	Bangladesh	4131	28	-	19	19	-	-	0	-	-	98	-	-	[96]
2020	2016–2019	Nepal	1367	3	-	2	3	-	-	0.2	-	-	87	-	-	[96]
2020	2016–2019	Pakistan	2093	83	-	82	82	-	-	66	-	-	95	-	-	[96]
2020	2012–2018	Pakistan	528	-	58	47	62	93	7	5	-	-	63		4	[97]
2024	2017–2023	Pakistan	3137	-	100	56–93	100–50	-	-	75–92	-	-	23–67	-	-	[98]
2024	2022–2023	Pakistan	5735	97	-	94	93	-	-	89	-	-	92	-	-	[99]
2024	2019–2021	Iraq	1471	77–82	-	6–7	13–7	-	12–17	93–89	-	16–6	21–12	-	4–3	[100]
2021	2010–2021	Etiopia	1837	-	-	89	-	78	-	6	-	-	20	-	-	[101]

Abbreviation: AM = ampicillin, AMX = amoxicillin, C = chloramphenicol, CX = cotrimoxazole, NA = nalidixic acid, CFM = cefixime, CRO = ceftriaxone, SSS = sulfonamides, TE = tetracycline, CIP = ciprofloxacin, STR = streptomycin, IPM = imipenem.

## 5. Prevention

The challenges of managing typhoid fever in endemic regions and among travelers have intensified global efforts to reduce morbidity and mortality through preventive measures, including World Health Organization (WHO)-approved vaccines and WASH interventions [1].

The effective control of typhoid fever requires a comprehensive surveillance system to evaluate the disease burden, alongside interventions such as access to clean water, improved sanitation, and the promotion of personal hygiene. Rapid diagnosis and treatment are essential to reduce disease transmission within communities. However, these efforts demand substantial financial investments and considerable time to implement them. Surveillance efforts include the early detection of cases and risk factors, outbreak monitoring, and the molecular and serological characterization of strains to track changes [110]. Enhancing access to safe drinking water, adequate sanitation, and proper wastewater disposal is essential to mitigate the disease’s impact in endemic areas. Concurrently, high standards of food hygiene and the timely treatment of acute cases and chronic carriers are crucial. Travelers can reduce their risk by practicing regular handwashing, drinking bottled water, avoiding street food, and receiving vaccinations prior to travel. Vaccination is particularly beneficial in reducing the disease incidence among infants, children, young adults, and food handlers in endemic regions [111,112]. In Western Europe and North America, typhoid fever cases have significantly declined due to the implementation of municipal water treatment, dairy pasteurization, and measures to prevent the human fecal contamination of food. Comparable decreases have been observed in parts of Latin America and Asia, driven by economic growth and improved water and sanitation systems [112]. The increasing incidence and outbreaks of typhoid fever underscore the urgent need for preventive and control strategies, including innovative vaccination programs and public health policies [113]. Vaccination offers an effective short-term solution [108], with *S.* Typhi vaccines demonstrating success when combined with measures like handwashing, water treatment, and hygiene improvements. Vaccination efforts should also include health education campaigns, WASH enhancements, and training for health workers in disease diagnosis and treatment [114]. As *S.* Typhi becomes increasingly resistant to antibiotics, vaccination is emerging as a crucial, cost-effective, and practical method of prevention, particularly in endemic regions and outbreak situations. The optimal strategy depends on local epidemiological factors, the identification of high-risk populations, and the region’s capacity to implement vaccination programs [111]. Significant progress has been made in understanding the global burden of typhoid fever through improved disease incidence studies and advanced modeling techniques. In 2017, the WHO Strategic Advisory Group of Experts on Immunization (SAGE) recommended the use of typhoid conjugate vaccine (TCV) for infants and children older than six months in endemic areas [115].

Since 2019, the Global Alliance for Vaccines and Immunization (Gavi) has supported the introduction of TCV in eligible countries. In March 2023, the WHO approved twoTCVs, both of which offer long-lasting protection and are safe for administration starting at six months of age [116].

### 5.1. Vaccines

The first vaccine developed against *S.* Typhi was an inactivated whole-cell vaccine, introduced over a century ago. Just like the live-attenuated oral vaccine Ty21a (Ty21a) and the Vi capsular polysaccharide vaccine (Vi-CPS), this vaccine was designed to target *S.* Typhi but was not implemented on a national scale. Its introduction in 1896 represented a major advancement, with extensive use by the British and American armies significantly reducing cases of typhoid fever and related deaths. Among the three types of vaccines developed for *S.* Typhi, the inactivated whole-cell vaccine proved to be the most effective, achieving a cumulative efficacy of 73% over three years. However, its high reactogenicity led to its withdrawal from widespread use, as these side effects were deemed acceptable for military personnel but unsuitable for general population immunization [117].

**Figure 2:**
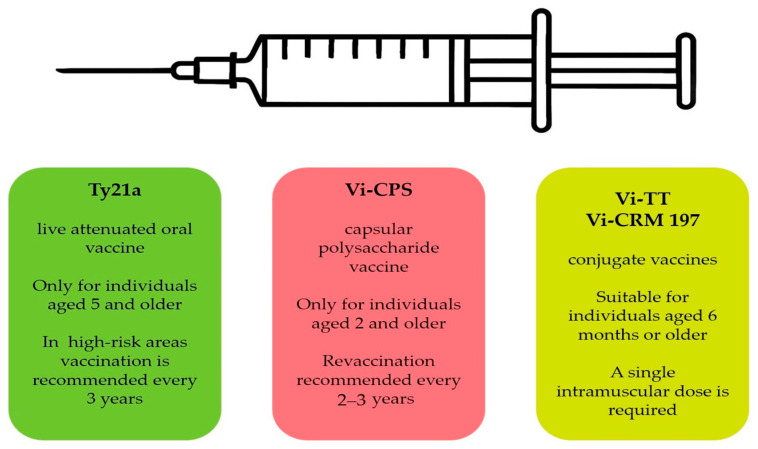
Overview of *Salmonella* Typhi vaccines.

Currently, three licensed vaccines are available for typhoid fever: the TCV, the Ty21a vaccine, and the Vi-CPS vaccine. Since 2008, the WHO has advocated for the use of *S.* Typhi vaccines in endemic countries. Despite this, the uptake of the Ty21a and Vi-CPS vaccines has been limited due to challenges such as the number of doses required, the short duration of protection, and insufficient funding from Gavi. TCVs address these limitations by offering significant advantages, including suitability for children under two years of age, aligning them with routine immunization programs [118] (Figure 2).

Infection with *S.* Typhi does not provide long-lasting immunity, and reinfections may occur weeks or months after an initial episode. These reinfections can result from either reactivation of the infection or reinfection from external sources, sometimes involving a different haplotype. Research has demonstrated that a variety of vaccines, including those using live-attenuated bacteria and Vi antigens, confer protection. Live vaccines lacking Vi expression (e.g., Ty21a) suggest the presence of both Vi-dependent and Vi-independent protective mechanisms. While immune responses to Vi are protective and involve mechanisms like complement-mediated killing and opsonization, their exact role in protection remains unclear [3]. Widely available typhoid vaccines, such as Ty21a and Vi-CPS, are not recommended for children under two years of age [118,119]. The Vi-CPS vaccine is indicated for children aged two years and older due to its low immunogenicity in younger children. Similarly, the oral Ty21a vaccine is recommended only for children aged five years and older due to its poor tolerance in younger age groups. In contrast, TCVs can be administered from infancy, as they include carrier proteins that induce T-cell-dependent immune responses. Immunogenicity studies indicate that TCVs generate comparable or higher anti-Vi IgG titers than Vi-PS, demonstrating a good immunogenicity and tolerance in children as young as six months. These next-generation vaccines are currently available internationally, primarily in India [119].

#### 5.1.1. Ty21a

Ty21a, the first live-attenuated oral vaccine against *Salmonella*, is available as enteric-coated liquid capsules (e.g., Vivotif^®^, produced by Crucell and PaxVax, Berne, Switzerland). It was developed in Switzerland through chemical mutagenesis of the wild-type Ty2 strain of *S.* Typhi [114,120]. Initially approved in Europe in 1983 and in the USA in 1989, it is recommended for adults and children over 5 years of age, with the age group 5–15-year being the most vulnerable to the disease. In high-risk areas, vaccination is recommended every 3 years, while for travelers from non-endemic to endemic areas, annual revaccination is advised [120].

The live-attenuated oral Ty21a vaccine stimulates local, cellular, and systemic immunity simultaneously, which is a unique triple-action effect not seen in parenteral vaccines [120,121]. However, the vaccine’s efficacy is limited to 50–70% of the population [121,122,123,124]. Its main drawbacks include reactogenicity and the need for multiple doses to achieve a robust immune response. In cases of ongoing exposure, immunization must be repeated every five years [121]. On the other hand, oral administration of this live-attenuated vaccine provides additional benefits compared to injectable Vi subunit vaccines, including longer-lasting protection, the induction of immunological memory, and a more natural immune response [125].

#### 5.1.2. Vi-CPS Vaccine

The Vi-CPS vaccine contains purified capsular polysaccharide Vi antigen and is available as a single-dose injectable for individuals aged 2 years and older [113,124]. It is not approved for children under 2 years due to its low immunogenicity. As a T-cell-independent antigen, it does not generate immunological memory or allow for an amplification of the immune response through repeated vaccinations [124]. Like other polysaccharide vaccines, there is no booster effect, and immunity lasts only a short time, with revaccination recommended every 2–3 years [113]. The vaccine’s efficacy is 55–59% over 2 years and 55% over 3 years, with no significant variation between studies [124,126,127].

#### 5.1.3. TCV Vaccine

In 2017, the first TCV vaccine, Vi-TT (Typbar TCV, Bharat Biotech International Ltd., Medchal, Malkajgiri District, Telangana State, India) [119], and a second TCV vaccine, Vi-CRM 197 (TYPHIBEV, Biological E, Medchal, Malkajgiri District, Telangana State, India), which combines Vi with a non-toxic mutant of diphtheria toxin [128], were licensed by the Drugs Controller General of India (DCGI) and prequalified by the WHO in 2020 [129].

Studies have shown promising results for the efficacy and safety of TCV vaccines in endemic regions, demonstrating an efficacy of 75–88% in children under 5 years or 5 years and older, which is superior to older vaccines [130,131]. A single intramuscular dose is required for all TCVs [132]. A long-term study conducted on Pakistan’s children under 10 years of age, vaccinated with a single dose of TCV, found that 96% developed antibodies 4–6 weeks post vaccination, and 76% maintained positive antibody levels for 4 years, showing a durable immunological response. The seroconversion rate at 4–6 weeks was higher in children under 2 years (99%) than in those aged 2–5 years (96%) and 5–10 years (93%). However, antibody decline was faster in children under 2, with only 63% maintaining antibodies after 4 years, compared to 79% in children aged 2–5 years and 83% in those aged 5–10 years [133].

A mathematical model showed that achieving an 80% vaccination coverage could prevent 21,449 cases within 10 years of the vaccine’s introduction, representing a 44% reduction compared to no vaccination [134].

TCV has proven effective in preventing *S.* Typhi infection, both in response to outbreaks and as part of routine immunization. It also has the potential to curb the spread of drug-resistant strains, particularly in light of the rise of XDR typhoid strains already reported in Pakistan [131,135]. However, additional research is required to assess TCV’s impact on AMR, long-term efficacy, and the role of booster doses in young children. Further studies should also investigate reductions in hospitalization rates, associated morbidities, and the overall *S.* Typhi incidence as indirect indicators of the protection offered by TCV [131].

An appealing approach for developing combination vaccines against *Salmonella* is to base them on licensed TCVs. For compatibility, these vaccines should consist of components suitable for parenteral administration [136].

#### 5.1.4. Paratyphoid Vaccine

Currently, there is no specific vaccine for paratyphoid fever, although the similarities between *S.* Paratyphi and *S.* Typhi suggest that developing such a vaccine is possible [137]. Bivalent vaccines (targeting *S.* Typhi and *S.* Paratyphi) and tetravalent vaccines (which include *S.* Typhimurium, *S.* Enteritidis, *S.* Typhi Vi, and *S.* Paratyphi A) show promise as potential solutions for preventing paratyphoid fever and other *Salmonella* infections, particularly in endemic regions. These vaccines have demonstrated safety and immunogenicity in preclinical studies [136,137,138,139,140]. Recent research indicates that tetravalent vaccines developed using MAPS (multiple antigen presentation system) technology could simplify vaccination in endemic areas, but additional studies are needed to confirm their protective efficacy [140].

## 6. The Role of Sequencing in Typhoid Fever Prevention

Advancements in whole genome sequencing (WGS) technologies have made it an ideal tool for surveillance by providing precise, high-resolution data about the genotype of a bacterium. This method allows for the differentiation of strains that exhibit identical resistance phenotypes but are driven by different mechanisms. WGS can also help uncover the mechanisms behind antibiotic resistance in drugs not typically tested or in cases where AMR mechanisms are not well understood [141].

Breakthroughs in human immunology and structural biology have enabled the development of vaccines against various microorganisms based on molecular information obtained from WGS, without the need to cultivate the pathogen. By monitoring resistant strains and integrating WGS data into the development of new treatment strategies and vaccination campaigns, the spread of resistance can be curtailed [142]. Thus, WGS in *S.* Typhi research has enabled a more detailed understanding of the pathogen’s transmission and evolution [142,143]. With the reduced costs of genome sequencing technology in recent years, WGS has become more accessible and is now considered the most advanced method for identifying infections, as endorsed by international health authorities [144].

WGS has also been used to estimate molecular evolution rates, an important tool in epidemiological studies. This has revealed that *S.* Typhi and *S.* Paratyphi A exhibit lower substitution rates (1.78 × 10^−7^–8.02 × 10^−8^ substitutions/year/site) compared to generalist serovars such as *S.* Kentucky and *S.* Agona [142,143].

Genomic studies of *S.* Typhi have revealed significant genetic diversity and the widespread distribution of different variants, notably the H58 haplotype, which accounts for 47% of a global sample of 1832 isolates. The H58 haplotype, often associated withAMR, has spread from South Asia to Southeast Asia, West Asia, East Africa, and Fiji [145]. The Global Typhoid Genomics Consortium (GTGC) conducted a meta-analysis of 13,000 *S.* Typhi genomes from 110 countries, supporting previous findings and highlighting the emergence of distinct drug-resistant strains in various regions. Resistance to ciprofloxacin was particularly widespread, accounting for over 85% of cases in South Africa. By 2020, around 70% of *S.* Typhi strains in Pakistan were highly drug-resistant, though these variants have not spread significantly to other regions. Additionally, strains resistant to both ciprofloxacin and ceftriaxone have been identified, along with several azithromycin-resistant variants emerging in South Asia [146].

Genotype exchange has been observed between Bangladesh and neighboring South Asian countries (India, Pakistan, and Nepal), indicating the circulation of these strains across the region. Since 2011, the prevalence of the H58 genotype in this area has decreased, while there has been an increase in the diversity of non-H58 genotypes, particularly the 3.3.2 group, which has split into two localized subgroups (3.3.2.Bd1 and 3.3.2.Bd2). Additionally, there has been a rise in fluoroquinolone-resistant strains linked to mutations in the *gyr*A gene [147]. Using WGS, *S.* Typhi strains circulating in Kenya were studied, revealing that most belong to the 4.3.1 genotype (formerly the H58 haplotype) and carry mutations in the *gyr*A gene that confer quinolone resistance. These strains were related to those found in East African countries (Rwanda, Tanzania, Uganda) and a South Asian country (India) [148]. In Chile, 11 distinct genotypes of *S.* Typhi have been identified, with the most common being 2, 3.5, and 1.1 [149].

Phylogenetic analysis using WGS data has become the gold standard for determining the relationships between *S.* Typhi strains. WGS is also instrumental in genotype assignment, plasmid identification and characterization, gene content and homology comparisons, and the detection of molecular factors contributing to antibiotic resistance and virulence [149]. Furthermore, WGS is valuable for detecting *Salmonella* clusters, which is crucial for identifying outbreaks and informing epidemiological responses [148].

## 7. Research Gaps

Although substantial progress has been made in identifying key virulence factors of *S.* Typhi (such as the Vi capsule and typhoid toxin), the precise mechanisms by which the bacterium evades the immune system and causes chronic infections remain poorly understood.

The rapid emergence of MDR and XDR *S.* Typhi strains has surpassed research efforts to uncover the molecular mechanisms of resistance. There is a need to investigate how resistance genes spread within bacterial populations, and the environmental or clinical conditions that promote their dissemination. Chronic carriers of *S.* Typhi are crucial for the persistence of the disease, yet the factors leading to chronic carriage in some individuals are not well understood. Moreover, strategies for eliminating carriers without over-reliance on antibiotics, which may drive further resistance, remain elusive.

It is also unclear which specific immune responses, such as antibody titers or T-cell immunity, correlate with long-term protection against *S.* Typhi, limiting the ability to improve vaccine designs or predict their efficacy across different populations. Current vaccines, including Ty21a and Vi polysaccharide, offer only partial protection and have limitations, such as a reduced efficacy in young children and variable effectiveness across regions. Research on the longevity of immune responses from these vaccines and the potential need for booster doses remains limited, as does our understanding of their performance against MDR and XDR strains, which may differ in virulence and immune evasion.

Vaccine efficacy also varies across regions and populations, likely due to genetic differences, environmental factors, or co-infections. Further research is needed to explain why some vaccines perform better in certain areas and to explore how factors like malnutrition, HIV, or other infections impact vaccine effectiveness.

## 8. Future Directions

Research should focus on the molecular mechanisms of *S.* Typhi’s virulence, particularly host–pathogen interactions, using advanced techniques like CRISPR-Cas and single-cell sequencing to uncover immune evasion and chronic infection factors. The gut microbiome’s role in infection susceptibility and vaccine response also warrants further investigation.

Alternative therapies, such as bacteriophages or antimicrobial peptides, are needed to reduce antibiotic usage. Developing adjuvants that enhance immune clearance without antibiotics is another promising area. Strategies to identify, monitor, and treat chronic carriers through immunotherapies or novel antimicrobials should be prioritized, along with biomarker research for the early detection of asymptomatic carriers.

Next-generation vaccines, including conjugate and live-attenuated vaccines, should aim for strong, long-lasting immunity, especially in young children and against MDR/XDR strains. Identifying immune correlates of protection will improve vaccine development and efficacy monitoring. Population-based studies are essential to assess vaccines’ performance across diverse environments and genetic backgrounds, considering factors like malnutrition, HIV, and co-infections.

Efforts to integrate vaccination into public health systems, especially in endemic areas, should address logistical, economic, and cultural barriers. Optimizing vaccine schedules, boosting herd immunity, and developing thermostable vaccines that do not require cold-chain storage would improve accessibility in low-resource regions. Novel delivery methods, such as microneedle patches or inhalable vaccines, could enhance administration and compliance.

## 9. Materials and Methods

This narrative review is based on 149 studies selected following an extensive literature search conducted using established databases PubMed and Google Academic. Relevant studies were identified using a combination of search terms “*Salmonella* Typhi”, “virulence factors”, “antibiotic resistance”, “typhoid fever”, “prevention”, and “typhoid vaccine”. The search was refined using Boolean operators “AND” and “OR” to enhance precision.

Selection criteria included ensuring alignment with our central research question—“Is *Salmonella* Typhi a public health threat, and what is the role of vaccination in combatting typhoid fever?”—as well as publication year, study type, and quality of findings. Searches were conducted in PubMed and Google Academic using Boolean operators to refine the results. Following initial searches, articles were excluded based on duplication, publication date, and title relevance. A subsequent abstract screening led to further exclusions due to relevance, accessibility, or topic misalignment. A full-text review then eliminated some articles due to methodological inconsistencies, quality concerns, or language barriers.

## 10. Conclusions

Typhoid fever continues to be a major public health challenge, particularly in low- and middle-income countries with inadequate sanitation and limited access to clean water. Effective control and prevention require a multifaceted approach, including large-scale clinical studies to identify high-risk communities and develop targeted care strategies. The rising antibiotic resistance of *S.* Typhi highlights the urgent need for preventive measures, with vaccination emerging as a critical component, along with classical methods regarding water quality, hand hygiene, etc. Strengthening existing vaccination programs and developing new strategies tailored to regional risks are essential. The global genomic surveillance of *S.* Typhi is crucial for tracking the spread and genetic diversity of the pathogen, particularly in relation to AMR. Advancements in WGS provide valuable insights into the pathogen’s evolution, host adaptation, and the emergence of new strains. Addressing research gaps and fostering global collaboration is essential to reducing the burden of typhoid fever and improving vaccine strategies worldwide.

## Figures and Tables

**Table 1:** Typhoid toxin evolution, structure, synthesis and export.

Characteristics	Description
Evolution of the toxin	This toxin appears to have evolved by merging the functionalities of two exotoxins: cytolethal distending toxin (CDT) and pertussis toxin
Structure	AB-type bacterial toxin family, comprising an enzymatic subunit “A” and a receptor-binding subunit “B”, resulting in an A_2_B_5_ structure
	The toxin forms a pyramid, with PltB at the base, PltA at the center, and CdtB at the apex
Synthesis and export	Toxin is produced exclusively by intracellular *S.* Typhi strainsExport to the extracellular environment is mediated by *Salmonella*-containing vacuoles (SCVs)

## Abbreviations

The following abbreviations are used in this manuscript:

TCV	typhoid conjugate vaccine
Ty21a	live-attenuated oral vaccine Ty21a
Vi-CPS	Vi capsular polysaccharide vaccine
AM	ampicillin
AMX	amoxicillin
C	chloramphenicol
CX	cotrimoxazole
NA	nalidixic acid
CFM	cefixime
CRO	ceftriaxone
SSS	sulfonamides
TE	tetracycline
CIP	ciprofloxacin
STR	streptomycin
IPM	imipenem
WGS	whole genome sequencing
GBD	Global Burden of Disease
CDT	cytolethal distending toxin
SCVs	*Salmonella*-containing vacuoles
LPS	lipopolysaccharide
NTS	non-typhoidal *Salmonella*
D-GalNAcA	N-acetylgalactosaminuronic acid
SPI-7	*Salmonella* Pathogenicity Island-7
MDR	multidrug resistance
AMR	antimicrobial resistance
WASH	water, sanitation, and hygiene
XDR	extensively drug-resistant
WHO	World Health Organization
SAGE	Strategic Advisory Group of Experts on Immunization
Gavi	Global Alliance for Vaccines and Immunization
MAPS	Multiple Antigen Presentation System
GTGC	Global Typhoid Genomics Consortium
T3SS	III secretion system
